# An Assessment of the Biological Significance of a Visual Clutch Staging Scheme for Ovigerous Female American Lobster (*Homarus americanus*)

**DOI:** 10.3390/ani13243856

**Published:** 2023-12-15

**Authors:** Marthe Larsen Haarr, Erin Hope Miller, Julien Gaudette, Rémy Rochette

**Affiliations:** 1 Biology Department, University of New Brunswick Saint John, Saint John, NB E2L 4L5, Canada; marthe.haarr@unb.ca (M.L.H.);; 2 St. Andrews Biological Station, Department of Fisheries and Oceans Canada, St. Andrews, NB E5B 0E4, Canada

**Keywords:** lobster, *Homarus americanus*, embryonic development, clutch staging

## Abstract

**Simple Summary:**

The clutches of berried female lobsters can be classified into four stages based on appearance: black with tightly packed eggs (stage 1), brown (stage 2) or orange (stage 3) with loosely packed eggs (stage 3), and hatching (stage 4, clutch “mossy”). This scheme has been used for decades, yet precisely how it relates to the biology of the developing eggs has not been investigated. We kept berried females in aquaria and monitored both the appearance of their clutches and investigated eggs closely by picking ten at regular intervals and inspecting them under a microscope. Our findings suggest additional criteria to the staging scheme to increase its accuracy. Firstly, clutches should be inspected closely enough to note whether individual eggs are uniform in colour or two-toned, which indicates how much yolk is present. Secondly, stage 3 clutches should include all eggs that contain ≤1/3 yolk to capture the period of rapid spring-time development leading to hatch. Thirdly, the presence of pre-zoea should be used to indicate a hatching (stage 4) clutch as they do not turn “mossy” (empty egg casings and adhesive substance visible) until late in the hatching process. Pre-zoea are newly hatched embryos not yet moulted to larvae and recognizable by their oval shape.

**Abstract:**

Qualitative visual clutch staging is a useful tool for rapidly and non-invasively assessing the developmental stage of American lobster, *Homarus americanus*, embryos. While such a scheme has been used in fisheries monitoring strategies in Canada since the 1980s, the biological relevance of its four visually distinguishable stages is poorly understood. We conducted a laboratory experiment in which 10 ovigerous females were housed and the development of their embryos regularly assessed, both qualitatively and quantitatively, from November until hatching in July/August. We confirmed the biological relevance of the qualitative staging scheme by showing clear quantitative differences in the duration and rate of embryonic development of stages 2–4 (stage 1 was not assessed as the precise spawning date was unknown). Stage 2 represents winter–spring “dormancy”. Stage 3 represents a shorter period of rapid development preceding hatch. Stage 4 represents hatching. We also recommend some improvements to the qualitative staging scheme, specifically (1) adding criteria related to the portion of eggs that are occupied by yolk to increase the accuracy of staging, (2) slightly redefining stage 3 to ensure it encompasses the full period of rapid embryonic development pre-hatch, and (3) adding the presence of pre-zoeae as a key indicator of hatching to avoid the misclassification of clutches in the early stages of hatching or those that are completely spent but still have adhesive substance.

## 1. Introduction

The American lobster *Homarus americanus* (hereafter, lobster) supports the most socio-economically important fishery on the east coast of North America, employing well over 20,000 licenced harvesters [1,2,3] and generating landings valued at nearly CAD 3 billion in 2021 [2,4]. The fishery is “effort controlled”, including restrictions on licences, gear, and seasons, regulations concerning minimum legal size that (generally) relate to female size at maturity, and a complete ban on landing ovigerous females [5]. The protection of ovigerous female lobsters is actually one of the oldest fishery regulations in Canada, dating back to the early 1870s [6].

Reproduction in female lobsters is typically considered a two-year process in which a female moults and mates in one summer, stores the sperm in her seminal receptacle until the following summer when she spawns and fertilizes her eggs, and then carries the fertilized eggs/embryos under her abdomen for nine to twelve months before hatching and releasing larvae during the third summer [7]. Gonadic, embryonic, and larval development all vary positively with temperature, within tolerance limits [8,9,10,11,12]. Consequently, there is considerable potential for lobster reproductive phenology to be affected by interannual variation in the thermal conditions experienced during different parts of the reproductive cycle. Upper water (<75 m) temperature has been increasing globally on average by approximately 0.1 °C per decade since 1970 and by 1 °C per decade in the northwest Atlantic [13,14,15,16,17], and there is evidence that females may have begun hatching their eggs earlier in the spring in response to increased fall temperatures several months before hatching occurs [18]. Given decreases in settlements of post-larval lobsters in some areas, and the relationship between settlement rates and subsequent recruitment to the fishery and landings [19,20], reproductive phenology should be monitored for this commercially important species.

Most Lobster Fishing Areas (LFAs) in Canada have a spring fishing season and are closed to fishing during later parts of the summer [1,5]. There are different motivations behind the summer closures, including protecting lobsters during moulting, egg laying, and hatching [21]. Although ovigerous females are always protected, their handling during the fishery can result in significant egg loss, particularly when embryos are in the late stages of development [22,23]. Monitoring of spring-time clutch condition and development, including assessing how these might be changing with rising water temperature, is therefore highly relevant to the fishery. This monitoring can also provide indications of interannual variation in hatching time, which may impact larval dispersal and survival by influencing food supply and the environmental conditions they experience. A temporal mismatch between lobster larvae and their prey is particularly likely if the hatching time of lobster larvae is influenced by temperatures in the fall, many months before hatching, given that these fall temperatures are unlikely to have the same impact on zooplankton prey with shorter life cycles [18,24,25,26].

A scheme for visually classifying the developmental stage of ovigerous females’ clutches has been included in fisheries monitoring strategies in the southern Gulf of St. Lawrence since the 1980s [27] and has been used by many groups in other regions, including in a recent large collaboration that spanned most fishing areas in Canada [28]. This staging scheme allows for a rapid and non-invasive assessment of clutch developmental stage in situ, on a scale from 1 to 4, based primarily on changes in color related to embryo development. Clutch colour has been used to indicate embryonic development stage for other decapods as well, such as the red deep-sea crab [29]. At stage 1, an American lobster clutch consists of newly spawned eggs that appear black or olive green in colour with no embryo visible to the naked eye. At stage 2, the eggs are further developed, and the clutch is lighter in colour, usually a shade of brown, with the embryos’ eye spots visible within the eggs. At stage 3, embryos are well developed and closer to hatching, with the overall clutch appearing tan to orange in colour. At stage 4, embryos are in the process of hatching as “pre-zoea”, and empty egg casings and adhesive material are found on the abdomen (i.e., the clutch appears “mossy”). During stage 4, embryos/pre-zoea will hatch over several days to weeks [30]. In the early years of the monitoring program in the southern Gulf of St. Lawrence [27], stages 3 and 4 were not distinguished but rather grouped together as “clutches with well-developed embryos”; all four categories were distinguished beginning in 2004.

The developmental staging of embryos to predict hatching via the inspection of eggs under a microscope is used in current research, e.g., [31,32,33], but the visual clutch staging scheme is also still commonly used as it provides a less invasive and more rapid assessment that can readily be made during fishing activities. Although this classification scheme has been in use for over 40 years in Canada, there are no quantitative estimates of the characteristics of each stage, such as their duration and relative rates of embryonic development. Such estimates would enhance the information conveyed by these monitoring data and hence their usefulness; they could, for example, better inform us about the imminence of hatch and/or the duration of the hatch period. We therefore conducted a laboratory study with the objectives of (i) confirming the biological relevance of the stages in relation to the monitoring of hatch and (ii) determining whether clutch descriptions could be improved to ensure this relevance and increase staging consistency. Clutch development was observed from late fall through to hatching the following summer, and we quantified the duration and embryo development rate of clutch stages 2–4. We did not include stage 1 clutches in the analyses as stage 1 precedes the winter period of minimum growth (see [11] for why this is not a true diapause period) and have little to no potential to forecast the next larval release period.

## 2. Materials and Methods

Pre-ovigerous female lobsters were collected during a trawl survey near Grand Manan and the Wolves in the southwest Bay of Fundy, Canada, on 5 July 2013 and brought to the Department of Fisheries and Oceans Canada St. Andrews Biological Station. On November 20th, we selected 10 females (96–145 mm carapace length) that had spawned in the lab in September, and we housed them in individual floating crates in large flow-through holding tanks with a natural photoperiod and twice-weekly food rations (primarily herring). The water temperature followed the ambient seasonal temperatures of the nearby cove from which water was pumped, dropping from 13 °C in September to 1 °C in February and March and then increasing to 11–14 °C during the period of hatching in July and August.

Clutch stage and embryonic development were assessed from 20 November 2013 to the point at which all females had completed hatching, with the last sampling on 26 August 2014. Clutch stage was qualitatively assessed based on the clutch staging scheme described above, and embryo development status was quantitatively assessed using the Perkins Eye Index (PEI), which is the mean of the maximum length and width of the oval-shaped embryo eye [8]; the PEI is proportional to embryonic size, and it provides a proxy for embryo development status [8,11,34]. The average PEI of the 10 clutches (10 embryos per clutch) ranged from 255 to 415 µm (44–72% development per Helluy and Beltz, 1991 [34]) at the start of the study. Qualitatively, all these clutches were in stage 1 at this point as eye spots were not visible to the naked eye (i.e., only discernable when individual eggs were placed under a dissecting scope), and the eggs appeared solid black/dark green and were tightly packed.

On each sampling date, clutch status was visually assessed, and a small sample of eggs (10–20 from 3–4 haphazard locations within the clutch) was taken from each female and preserved in 65:35 ethanol/glycerol; the PEI was later measured on a sub-sample of 10 embryos haphazardly chosen from each of these females. All females were sampled more extensively in April, prior to the onset of stage 3, to determine whether there were differences in embryo development status based on their location in the clutch; 80 eggs were systematically collected from different positions within the brood (different combinations of each of the four pleopod pairs, the left and right sides, the surface and bottom of the egg mass, and the periphery and centre of the eggs mass; n = 5 for each position). Intra-brood variability in embryo PEI was assessed by using the AIC to compare ANOVA models involving all possible combinations of potential clutch locations, including interaction terms and considering the female as a random variable [35]. The null model (i.e., no consideration of position within the clutch) obtained the lowest AIC score and 30% of the AIC weight. Consequently, egg position within the clutch was not considered further, and eggs were simply sampled haphazardly from different locations within each clutch.

To minimise the handling of females while still capturing changes in embryo development status over time, the females’ clutches and embryos were assessed increasingly frequently over the study period as the temperature and embryo development rate increased: monthly from November to March, every other week in April, weekly in May and June, and daily (visual staging) or every other day (egg samples) in July and August. This does mean, however, that our estimates of stage duration are less precise for clutches of stage 2 than those of stages 3 and 4 due to the less frequent sampling at the beginning than at the end of the experiment. However, considering the markedly longer duration of stage 2 compared to stages 3 and 4, the relative precision was similar and is inconsequential to the use of staging to assess hatch.

Although our analyses of embryo development confirm a strong biological basis for the visual staging scheme (see Section 3), the qualitative features of the staging scheme are obviously not perfectly distinct, and there are “transitionary periods” during which the clutches can be difficult to classify. By the second sampling date on 17 December 2013, all clutches were transitioning to stage 2. In terms of colour and packing density, they still most closely resembled stage 1, yet the embryos’ eye spots were discernable by the naked eye in most eggs. Giving weight to the visibility of eye spots, we considered clutches to be in stage 2 during this December sampling. The following month, all clutches clearly met all criteria for stage 2, and all embryos inspected had eyespots that were visible to the naked eye. There were similarly clutches later during development that exhibited an intermediate colour between the typical brown of stage 2 and the orange/tan of stage 3 and that we temporarily classified as stage 2.5 to allow for a further investigation of the best criteria to distinguish the two stages upon analyses of embryonic development rates.

The duration of clutch stages 2, 3, and 4, and the rate of embryonic development during each stage were compared using mixed model ANOVAs, with the clutch stage as a fixed-effect factor and the female as a random-effect block factor; in other words, we assessed variability among clutch stages after accounting for variability among females. The duration of each stage was assessed for each female as the most conservative and the most liberal estimates given that sampling was not continuous, and thus the precise dates of the end and onset of a stage were not known. The most conservative duration estimate was the number of days between the first and last dates on which a given stage was observed for a female. The most liberal estimate for a particular stage and female was the number of days between the last date the previous stage was observed plus one day and the first date the following stage was observed minus one day. The rate of clutch development during each stage was calculated as the slope of the mean clutch PEI (µm) over time, resulting in a metric of mean daily increment in embryonic eye diameter for each clutch stage and female.

## 3. Results and Discussion

**Figure 1:**
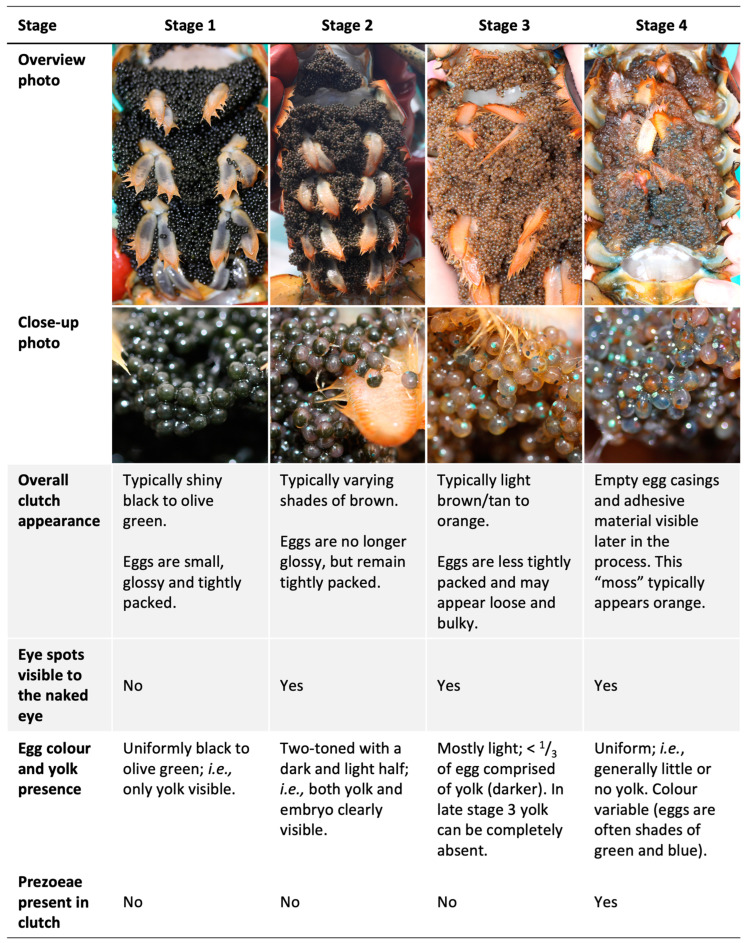
The visual clutch staging scheme for the qualitative assessment of the embryonic developmental stage of ovigerous female lobsters. Shaded parts of the table indicate criteria in the existing scheme, whereas unshaded parts are suggested additions to improve the accuracy and biological relevance of staging (see the text for explanations).

Our laboratory study confirmed that the visual clutch-staging scheme distinguishes biologically meaningful phases of embryonic development (Figure 1). The precise duration of stage 1 was unknown as the precise spawning dates were unknown. However, given that the females were known to have spawned in the lab sometime in September, this stage lasted approximately 2–3 months (from September to late November or mid-December) and is therefore only a rough indicator of spawning time. Based on previous studies, embryonic development rates are known to be rapid during this time relative to what they are in later fall and winter [8,11,34]. The smallest PEI measured in this study (156 µm) was larger than the smallest PEI (ca. 70 µm) measured in these earlier studies, which measured the first PEI earlier in the fall than we did. However, the first PEI values we measured are within range of the PEI values obtained at the same time of year in these earlier studies. Embryo eye spots are clearly present during a large portion of stage 1, even though the key distinction between this stage and stage 2 is the presence of said eye spots. However, stage I eye spots are not visible to the naked eye due to the large amount of dark yolk masking their presence.

**Figure 2:**
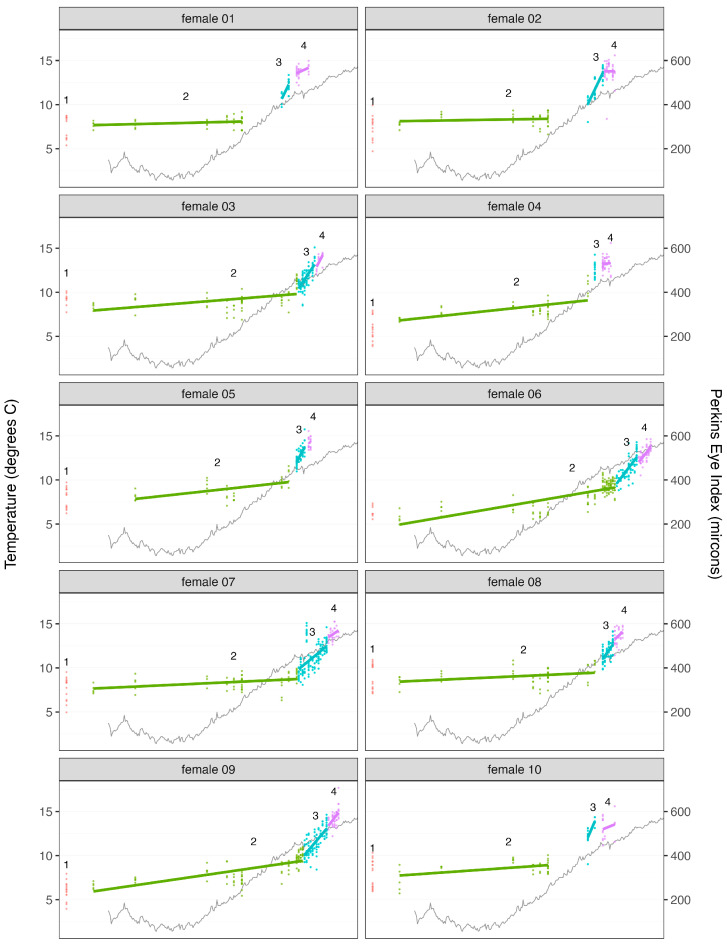
Graph showing (1) the mean daily temperature during the study (grey lines), (2) embryo development status (Perkins Eye Index) on each sampling date for each female (points), and (3) the minimum duration and mean embryonic development rate of each clutch stage (stage 2 embryos are shown by green points, stage 3 by turquoise and stage 4 by purple; see Figure 1 for descriptions) for each female. Clutches were in stage 1 (red) when we started the study, but we did not capture the duration of this stage.

Stage 2 is a relatively long period of very slow embryo development. All 10 clutches reached stage 2 by mid-December and remained in this stage throughout the winter and until May–July, for an estimated mean (±95% confidence interval) stage duration estimated conservatively at 182 ± 20 days (range among females: 149–216 days) and more liberally at 227 ± 7 days (range: 214–243 days) (Figure 2). On average, the embryos developed at a rate of 0.35 ± 0.16 µm day^−1^ during stage 2 (range: 0.07–0.72 µm day^−1^). These findings are consistent with the long period of slow or no embryonic development from late fall to spring reported by Perkins [8] and Gendron and Ouellet [11]. Our study further suggests that this dormancy period largely corresponds to stage 2 in the clutch staging scheme, while the periods of rapid embryonic development in fall and late spring/early summer correspond to stages 1 and 3, respectively.

Stage 3 is a much shorter phase of markedly more rapid embryonic development in summer (Figure 2) leading up to hatching. Development in this stage was in fact so rapid that not all females’ clutches were observed meeting all criteria for stage 3 (clutch orange/tan, eggs loosely packed) before proceeding to stage 4, nor were all clutches observed in the “transitionary period”, aka stage 2.5, where some characteristics of stages 2 and 3 were visible (primarily an intermediate colour). Qualitatively, the distinction between stages 2, 2.5, and 3 is likely related to the resorption of yolk by the embryo, which results in an overall lightening of the clutch’s visual appearance from brown to orange/tan. In the intermediary stage that we called 2.5, yolk does remain in the eggs, but the amount is reduced to less than approximately a third of the eggs’ volume, resulting in the overall appearance of the clutch being a lighter brown than stage 2 but not yet as light as stage 3. For females whose clutches were observed in stage 2.5, embryonic development was markedly more rapid than for stage 2 and similar to stage 3 (an average increase in PEI of 7.7 µm day^−1^ (3–15 µm day^−1^) for stage 2.5 and 11.0 µm day^−1^ (6–24 µm day^−1^) for stage 3). Consequently, we classified as stage 3 (rather than stage 2) those clutches that could not easily be distinguished visually as stage 2 or stage 3 clutches. Using this criterion, stage 3 clutches were observed from mid-June to late July, for a mean stage duration estimated conservatively at 13 ± 6 days (range among females: 6–28 days) and more liberally at 30 ± 12 days (range: 13–55 days) (Figure 2). On average, embryos in stage 3 clutches developed at a rate of 6.9 ± 1.9 µm day^−1^ (range: 2.5–10.5 µm day^−1^). i.e., approximately 20 times faster than during stage 2.

Stage 4 clutches, which are indicative of hatching, were observed from early July to late August for a mean stage duration estimated conservatively at 9 ± 2 days (range among females: 2–12 days) and more liberally at 11 ± 3 days (range: 5–19 days) (Figure 2). On average, embryos in stage 4 clutches developed at a rate of 2.9 ± 2.2 µm day^−1^ (range among females: −0.6–8.8 µm day^−1^) and hence at less than half the rate of embryos in the previous stage. This apparent slowing of embryonic development during hatching might in part be related to a “development hiatus” associated with the embryo’s last molt prior to hatch [34], but is also likely at least partly the result of an increasing number of the faster-developing/most developed embryos being “lost” over the course of the hatching period, leaving the slower/less-developed embryos from which to estimate mean development within the clutch as the hatch progresses.

The differences in the duration of clutch stages 2–4 were highly significant (F_2,18_ = 861.20, *p* < 0.0001 and F_2,18_ = 408.22, *p* < 0.0001, the most conservative and liberal estimates, respectively), as were those concerning their rate of embryonic development (F = 22.99, *p* < 0.0001), and neither of these varied significantly among females (stage duration more conservative: F_9,18_ = 0.46, *p* = 0.88; stage duration most liberal: F_9,18_ = 1.66, *p* = 0.17; embryonic development rate: F_9,18_ = 0.92, *p* = 0.53) (Figure 2).

The clutches of embryos experienced marked increases in temperature over the course of their development (Figure 2), and these increases were likely responsible for most of the changes we observed in embryo development rates and in our qualitative assessments of clutch stages. These increases in temperature likely contributed, in particular, to the most pronounced change we observed, i.e., the resumption of rapid embryonic development by stage 3 clutches following the “dormancy” of stage 2 clutches in the winter–spring, leading to hatching in late spring–summer. For example, the mean temperature at which a stage 2 clutch was observed varied from 3 to 5 °C among the 10 females, with a total range of 1.4 to 11.6 °C, in comparison to 10–12 °C and 10–13.3 °C for the stage 3 clutches. Similarly, both Perkins [8] and Gendron and Ouellet [11] observed very little to no development of embryos at temperatures below ca. 6–7 °C during winter and spring months. Nevertheless, the transition between stage 2 and 3 clutches was not entirely explained by water temperature as the maximum temperature at which a stage 2 clutch was observed varied between 6 and 12 °C among our 10 study females, which contributed to the initial detection of stage 3 clutches varying by as many as 29 days among these females.

Similarly, same-stage duration varied among females, even though these were held at the same temperature (Figure 2). This non-perfect relation between water temperature and development is also evident at the level of individual embryos from the same clutch, as the PEI of such embryos varied markedly at any one point in time (Figure 2), and for all females, despite the fact that these embryos were all likely spawned within fewer than approximately three to five hours of one another [9,36]. Perkins [8] similarly noted that embryonic development is not strictly tied to temperature as older embryos developed more slowly than younger embryos, even though the females were held in the same tank (spawning dates up to 7 weeks apart, but a range in total time from spawn to hatch of only 4.5 weeks), and Gendron and Ouellet [11] also observed later-spawning clutches continuing very slow development through the winter dormancy period to partially “catch up” to those spawned earlier. It should be noted that wild ovigerous females can undertake seasonal depth migrations that influence the temperature experienced by their embryos during winter [37,38], unlike the lobsters we held in the lab, which could impact the exact timing of the stages. Whereas such movements may affect the relative duration of the different clutch stages, these effects are likely relatively minor given the marked differences in stage duration noted in this study. But more importantly, such movements are unlikely to affect the visual appearance of the different clutch stages, which was our primary focus.

**Figure 3:**
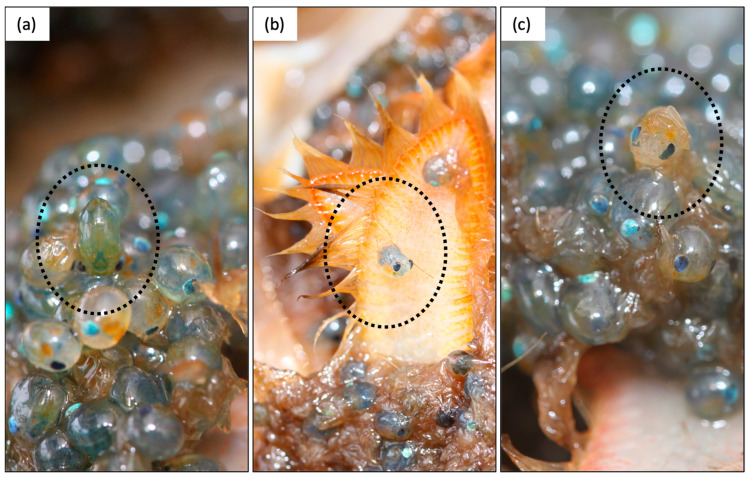
Photographs showing pre-zoeae in situ. With just a little experience so one knows what to look for, these are easy to spot in a clutch when present and can be used to reduce errors around the positive identification of stage 4 (hatching) clutches. (**a**) Note the slightly elongated and more oval shape of the pre-zoea relative to the embryos and the more pronounced bulging of the eyes, both occurring because the pre-zoea is no longer covered by the egg envelope. (**b**) A pre-zoea that has emerged from both the outer and inner egg envelopes, such that the telson is visible. Note again the bulging eyes. (**c**) A pre-zoea in the process of emerging from outer egg envelope, which is visible as a crinkled “skin”. See Helluy and Beltz (1991) [34] for detailed descriptions and photographs of pre-zoeae.

We propose three ways the qualitative staging scheme can be improved (Figure 1). Firstly, we recommend making the presence of pre-zoeae (newly hatched pre-larvae not yet moulted to stage I larvae [39]) the key defining criterion of stage 4 clutches (Figure 1) to increase the positive identification of stage 4 clutches in the early stages of hatching and to decrease the false identification of hatching soon after a clutch is fully spawned. Currently, the main criterion distinguishing stage 3 and 4 clutches is the absence/presence of “moss” (i.e., empty egg casings and adhesive material). However, in this study, we observed “mossy” clutches only after these had been hatching for many days, as evidenced by the presence of larvae in the female holding baskets. Pre-zoeae, on the other hand, were visible within a female’s clutch during the entire hatching period, including at the very beginning of hatch. Therefore, if the presence of “moss” is a necessary criterion for stage 4, clutches in the early stages of releasing larvae are likely to be misclassified as stage 3. Pre-zoeae are readily identified by their oval shape compared to eggs (Figure 3), because they have broken from the egg membrane, and including their presence as a criterion for stage 4 should increase the accuracy of clutch staging. We also recommend that “mossy” clutches in which no pre-zoeae or eggs are present not be classified as ovigerous as hatching is complete at this stage (alternately, this can be recorded as stage 5).

Secondly, we recommend the addition of criteria relating to the yolk content of individual eggs in each stage (Figure 1) to reduce the potential for observer bias. Yolk content relates to clutch colour, as this lightens with decreasing yolk remaining in the eggs. Stage 1 eggs contain so much yolk that they appear almost uniform in colour and mask embryo eye spots even after these are evident under a dissecting scope. Stage 2 eggs are clearly two-toned, with a light and a dark half, as yolk comprises roughly two-thirds to one-third of the eggs. Stage 3 and 4 eggs contain relatively little or no yolk (i.e., only a small darker patch visible constituting no more than one-third of the egg). Noting yolk content as discernable by the naked eye can aid decision making when stage classification may be ambiguous as clutches transition between stages. Clutches drastically change colour from stage 3 to early stage 4, becoming markedly darker because individual eggs turn variable dark colours, typically greens and blues, immediately before hatching [39]. In the early stages of hatching, before a clutch becomes “mossy”, this could therefore lead observers (particularly inexperienced ones) to erroneously classify early stage 4 clutches as stage 2 clutches, as the previously light-coloured orange or tan stage 3 clutch is now darker and may superficially resemble the darker brown appearance of a stage 2 clutch. Noting the yolk content of the eggs, however, would eliminate this potential error. While some embryos do hatch before depleting their yolk reserves [40], the difference between some to no yolk present is readily distinguishable from the clearly two-toned eggs of a stage 2 clutch, where approximately half the egg constitutes yolk. Noting yolk content also allows for correct stage classification in the event of clutch colour abnormalities, which can occur (M.L. Haarr, personal observation). Yolk classification is furthermore a very straightforward additional step during classification. One needs simply to inspect the clutch at close range and note how large a portion of the egg is dark (yolk) vs. light (see Figure 1), during which one can also readily check for pre-zoaea (first recommendation).

Thirdly, we recommend that the description of stage 3 be amended to ensure the inclusion of clutches that have only recently started rapid development following winter/spring dormancy which, based on the current scheme, can be classified as stage 2, as the distinction between stages 2 and 3 is mainly based on color (Figure 1). There is a period in early summer when the embryos have started developing rapidly following dormancy, but the overall appearance of the clutch is more similar to stage 2 (shades of brown) than to stage 3 (tan or orange) because the eggs still contain a fair amount of yolk (up to one-third of the egg). As the presence of yolk darkens the overall colour of the clutch, these clutches are less orange in appearance at this stage than more advanced stage 3 clutches, although they are still a somewhat paler brown than stage 2 clutches, the eggs of which are approximately half filled with yolk. The embryonic development rate during this early stage 3 (light brown clutch; yolk up to one-third of the egg) was rapid (mean increase in PEI of 7.7 µm day^−1^; range 3–15 µm day^−1^) and much more comparable to “mature” stage 3 clutches ($\bar{x}$ = 11 µm day^−1^; range 6–24 µm day^−1^) than to stage 2 clutches ($\bar{x}$ = 0.4 µm day^−1^). The current “cut-off” for how orange a clutch must be to be considered stage 3 is somewhat observer-dependent, although historically in the southern Gulf of St. Lawrence monitoring program, only the most orange clutches have typically been classified as stage 3 (M. Comeau, Fisheries and Oceans Canada, pers. Comm.), suggesting the exclusion of some clutches with embryos that have begun developing rapidly following winter dormancy. Broadening the definition of stage 3 clutches to include all those that are lighter brown as well as orange in colour, and with eggs comprising less than one-third yolk, would be biologically relevant as it would capture the entire period of rapid embryonic development preceding hatch. The previous recommendation of including egg yolk content in the stage criteria also enables this biologically relevant distinction of eggs in dormancy (stage 2, ≈1/2 yolk) and in rapid development (stage 3, ≤1/3 yolk).

## 4. Conclusions

This study demonstrates clear differences in duration and embryo development rates among stages of a non-invasive visual clutch-staging scheme commonly used to monitor the development and hatching of American lobster embryos, confirming the biological relevance of this scheme. To maximise the biological information conveyed and reduce potential classification errors, we recommend three easy-to-implement improvements to the scheme: (1) adding criteria related to the amount of yolk present in individual eggs rather than overall clutch appearance and colour alone, (2) redefining stage 3 to ensure it encompasses the beginning of rapid embryonic development following winter dormancy by including all paler (lighter brown/tan to orange) clutches with eggs containing ≤1/3 yolk, and (3) adding the presence of pre-zoeae as the key indicator of hatching (stage 4). Future research can utilise this scheme to rapidly and non-invasively elucidate spatial and inter-annual variability in larval release patterns in the lobster.

## Data Availability

Raw data will be made available through Rémy Rochette’s website (https://www.rochettelab.com, accessed on 1 December 2023).
